# Characterizing intersecting social determinants of health during pregnancy: a descriptive cross-sectional analysis from a northern New England health system

**DOI:** 10.3389/fmed.2025.1658735

**Published:** 2025-12-17

**Authors:** Alka Dev, Sophia E. Allen, Ilana Cass, Chelsey R. Canavan, Vinisha Velmineti, Daisy J. Goodman

**Affiliations:** 1 The Dartmouth Institute for Health Policy and Clinical Practice, Geisel School of Medicine at Dartmouth College, Lebanon, NH, United States; 2 Department of Obstetrics and Gynecology, Dartmouth Health, Lebanon, NH, United States; 3 Department of Population Health, Dartmouth Health, Lebanon, NH, United States

**Keywords:** social determinants of health, prenatal care, screening, rural, health equity, food insecurity, housing instability, social isolation

## Abstract

**Objective:**

We aimed to characterize the prevalence and co-occurrence of social determinants of health among pregnant individuals during prenatal care in a single rural-based health system in the United States.

**Context and case:**

This community case study describes the implementation and findings of universal screening for social determinants of health among all individuals initiating prenatal care at four OB-GYN clinics in a single health system between January 2022 and December 2023. Antenatal sites spanned both small urban and rural settings, with a subset of deliveries occurring at a rural, tertiary care medical center. Social determinants, including financial stress, food insecurity, housing instability, transportation issues, social isolation, and health literacy, were assessed using a screening tool embedded in the electronic health record (EHR). We used descriptive statistics and UpSet Plots to describe these determinants and their co-occurrence patterns in relation to patient characteristics.

**Findings:**

Among 2,222 pregnant individuals who completed screening, 16.7% reported at least one social determinant, and 7.8% reported two or more. Among patients who screened positive for only one determinant, the most common concern was social isolation (38.9%), followed by financial stress (27.8%). Among those with two or more determinants, the most common items were financial stress (75.1%), food insecurity (67.6%), and housing instability (56.1%). Combinations of food insecurity, housing instability, and financial stress affected nearly one in 10 patients.

**Conclusion:**

Social isolation emerged as a significant concern for non-urban pregnant women who had no other reported social determinants. However, determinants also co-occurred, particularly housing and food insecurity with financial stress. This descriptive analysis provides foundational data for future research examining associations between intersecting social determinants and maternal–infant health outcomes. Universal screening is critically important for identifying patients with high social risk.

## Introduction

It is well established that a woman’s social status, as measured by characteristics such as race, ethnicity, nativity, education, income, and employment, is associated with her perinatal health outcomes (1–3). Higher prevalence of social and economic risk factors, known collectively as the social determinants of health (SDOH), is consistently associated with increased risk of adverse pregnancy and birth outcomes. Lack of access to essential resources, including nutritious food, stable housing, reliable transportation, affordable utilities, childcare, healthcare, and safe living environments, also limits the ability to maintain reproductive health before conception and, therefore, further impacts the ability to have a healthy pregnancy (4, 5).

Food insecurity during pregnancy is linked to a range of perinatal complications, including iron deficiency, gestational diabetes, hypertensive disorders of pregnancy, preeclampsia, fetal growth restriction, low birth weight, preterm delivery, and stillbirth (6, 7). Potential mechanisms of action include poor nutritional quality, including low protein and high glycemic foods, inconsistent access to food, insufficient calories, and high levels of prenatal stress related to the inability to obtain sufficient food or to secure it reliably (6). Additionally, poor quality, unstable, or unaffordable housing during pregnancy correlates with lower birth weight, preterm labor and birth, and severe maternal morbidity (8–10). Factors such as overcrowded living conditions, inadequate heating during the winter or overheating in summer, exposure to mold and other respiratory irritants, and the stress of housing instability can all contribute to adverse birth outcomes.

Co-occurring SDOH can also amplify each other, creating complex layers of disadvantage (11). For example, poor nutritional quality during pregnancy resulting from the inability to purchase healthy foods is exacerbated by limited access to nutritious food sources in food deserts, or the lack of proper cooking equipment or a kitchen when housing is unstable. Interviews with prenatal care and postpartum patients in our rural area documented these intersecting experiences; among pregnant patients facing food insecurity, at least half also reported housing instability, including difficulty paying rent, precarious, unsafe, or overcrowded living conditions, and being evicted or forced to move (12).

The American College of Obstetricians and Gynecologists and the Joint Commission, the principle standards-setting and accrediting body in U.S. healthcare, recommend universal screening with previously validated screening tools to allow clinical staff to identify patients with SDOH who may be at associated higher risk for adverse health outcomes (13, 14). However, fewer than half of hospitals systematically screen for SDOH, despite the increasing feasibility of embedding universal screening in electronic health record (EHR) systems, and available tools that assess a wide range of social characteristics (15). The Centers for Medicare and Medicaid Services have further proposed mandating inpatient SDOH screening to outpatient settings, such as ambulatory clinics, by 2026. This expansion is intended to help providers understand the prevalence of social risk in their patient populations. In parallel with these recommendations, CMS has released new reimbursement frameworks for caring for high social risk patients (16).

Despite the well documented impact of unmet SDOH needs on perinatal outcomes, the role of universal screening on understanding social risk, and the extent of social risk due to overlapping needs among pregnant patients has not been well studied. The process of developing a universal, technology-based SDOH screening program for perinatal patients across our health system and its acceptability for both patients and providers has been reported previously (17). In the current study, we aimed to describe the prevalence and characteristics of patient-reported SDOH during pregnancy in our health system, as well as challenges inherent in assessing these through an EHR-embedded screening approach. We describe the setting and the conceptual framework which guided our study, followed by quantitative analysis of screening data and its implications for clinical practice and allocation of resources.

## Community context

### Setting

Dartmouth Health is an integrated health system with its hub at an academic medical center in the rural central part of the state of New Hampshire (NH). The health system is NH’s only tertiary maternity care provider, accepting high-risk obstetric patients from across the region. A recent community needs assessment conducted by the medical center revealed the extent of SDOH needs in our service area, finding that over half of the responders worried about out-of-pocket expenses (61%) and transportation (55%). Additionally, necessities like food and shelter (36%) and, to some degree, internet access (14%) were lacking, and people feared both stigma (21%) and discrimination (19%) upon asking for help (18). Complicating community social needs is the crisis in maternal health care access. Since 2000, 11 hospitals in the state have closed their labor and delivery departments, of which nine were in rural areas, representing more than 40% of the state’s birthing hospitals (19). These closures have led to increased driving time to labor and delivery services for patients and also increased demand at the remaining facilities in these “maternity care deserts,” typically encompassing lower-income and more rural communities (20). The hospital’s Department of Obstetrics and Gynecology (OB-GYN) includes an outpatient clinic at the academic hub, and three OB-GYN practices located in the state’s three largest cities (ranging in population size from 44,000 to 115,000). All affiliated practices share an EHR. In December 2021, in anticipation of national mandates for universal social risk screening of patients in both inpatient and outpatient settings, the OB-GYN department initiated SDOH screening of all pregnant patients receiving prenatal care at one of the four obstetric practices.

### Conceptual framework

Our approach was guided by two qualitative studies conducted by our research team with pregnant and postpartum patients receiving care in this same health system (12, 17). These studies involved semi-structured interviews with 40 individuals recruited from the same four OB-GYN clinical sites included in the current analysis, from 2020 to 2022. In interviews, patients indicated that significant financial stress stemmed from the relationship between securing housing and food and the inability to work due to pregnancy-associated morbidity. Transportation issues in rural contexts, such as not owning a vehicle and limited or nonexistent public transportation options, were also notable barriers to food access in areas where grocery stores were in larger towns only. Similarly, lack of internet access delayed the ability to apply for subsidized housing. Support from family members was important for addressing gaps (e.g., providing emergency shelter), but this support was unpredictable due to everyone’s financial instability, reflecting how spatial concentration of poverty means that people in local social networks may not be able to help when needed. Any support through the health system, however, was seen as consistent and welcomed, representing a potentially modifiable resource within otherwise constrained community environments. Fear of stigma associated with unmet social needs was mitigated to some degree by the involvement of community health workers, when available in the health system (17).

**Figure 1:**
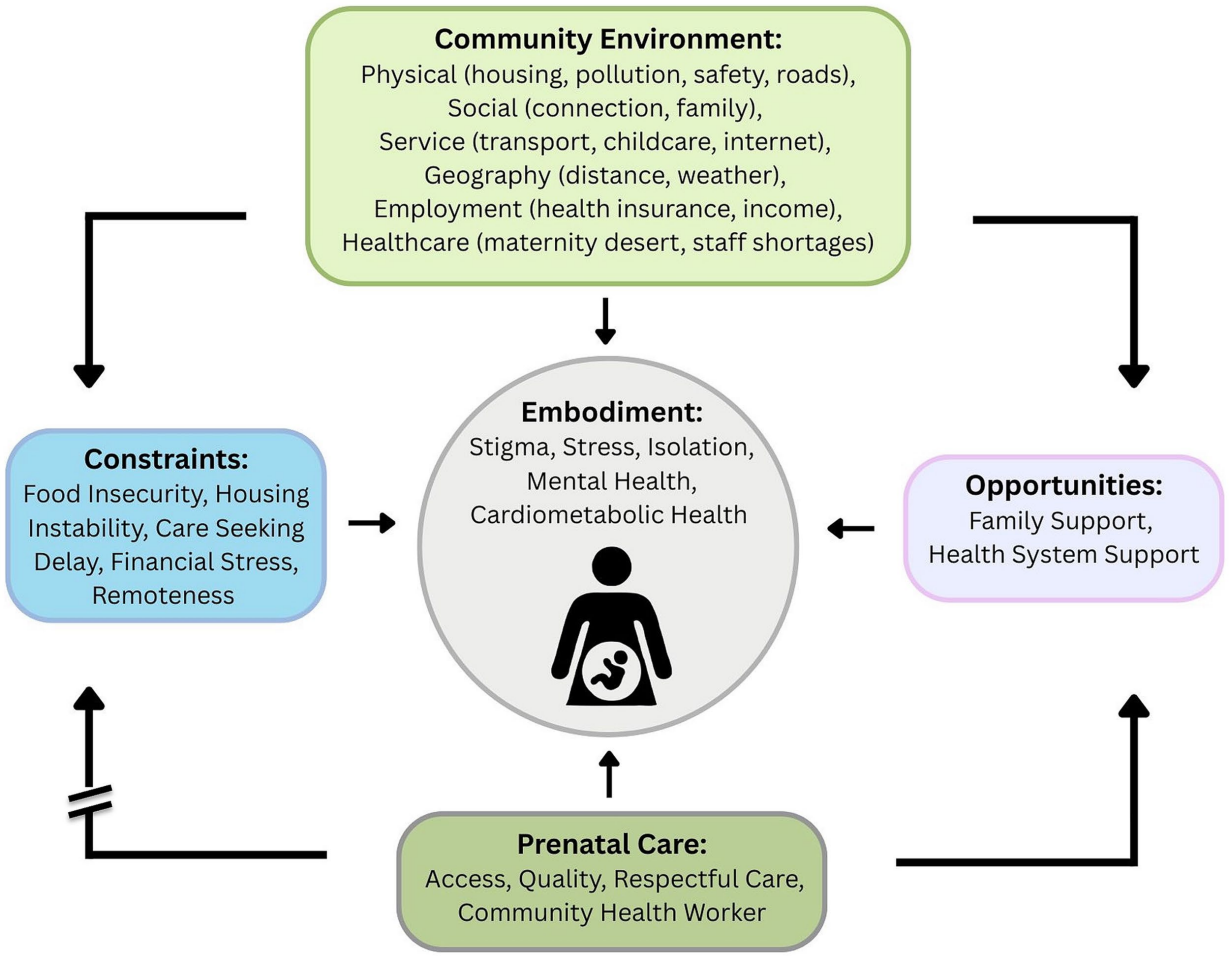
Conceptual model.

The qualitative perspectives on the lived experience of SDOH and the mechanisms linking SDOH, elicited through our qualitative work, informed the adaptation of Kramer et al.’s maternal health equity framework (21). Within this framework (Figure 1), community environments act as “risk regulators,” factors that constrain or enable individual behaviors, health access, and exposures. At the center is the embodied pregnant woman experiencing the biological manifestation of social experiences including stigma, stress, isolation, mental health concerns, and cardiometabolic health (22). The model shows how community environment factors (encompassing physical conditions like housing and pollution, social connections and family support, services such as transportation and childcare, geography including distance and weather, employment-related factors like health insurance and income, and healthcare access including maternity care availability and staff shortages) influence maternal health through multiple pathways. These environmental factors create both constraints, such as food insecurity, housing instability, care-seeking delays, financial stress, and remoteness, and opportunities, including family and health system support. The framework emphasizes that prenatal care characteristics (access, quality, respectful care, and community health worker involvement) directly impact maternal outcomes by disrupting constraints, attending to embodied health outcomes, and increasing healthy opportunities for pregnant people.

## Methods

### Study design

This is a descriptive cross-sectional analysis examining SDOH prevalence and intersectionality at a single point in time (first prenatal visit) among pregnant individuals in our health system. Our analysis focuses on SDOH needs reported at the initiation of prenatal care rather than over the course of pregnancy, making this a cross-sectional study. The participants were similar to those in our qualitative work in that they initiated prenatal care in our health system and were screened for SDOH.

### Measures of social determinants

SDOH were identified using a screening tool embedded in the EHR starting in December 2021 (23). Domains were drawn from the EPIC® standardized SDOH screening bank of 50 pre-validated questions, which aggregates questions from previously validated instruments and screening tools endorsed by the Centers for Medicare & Medicaid Services and the National Academy of Medicine (23), such as the 2-item Hunger Vital Sign from Children’s HealthWatch, which has demonstrated sensitivity of 97% and specificity of 83% for identifying food insecurity in diverse populations (24, 25).

The screening tool drew from an 11-domain bank and initially included 29 questions embedded in the patient EHR and administered via tablet or the patient portal to all OB-GYN patients at the first prenatal care appointment, 28 weeks of gestation, and the first postpartum visit. However, upon adoption by other clinical departments that subsequently initiated SDOH screening, the tool was reduced to 14 questions across the entire health system, which also affected the questions in the OB-GYN screener. Questions related to phone and internet access, safety, legal problems, and domestic violence were removed to reduce patient burden. To ensure consistency in our analysis, we included only those six domains that were consistently measured across the entire observation period from December 2021 through December 2023. While this modification limited our ability to assess other SDOH domains, it ensured the validity of temporal aggregation within our dataset.

All patients included in this analysis responded to questions from the six core SDOH domains that remained consistent throughout screening tool versions. Specifically:

- Patients screened December 2021 – May 2023: Responded to 29 questions covering 11 domains; our analysis extracted only responses from the six domains which were carried forward after revision.
- Patients screened June 2023–December 2023: Responded to 14 questions covering the same six domains; all responses were included.

This approach ensured that all patients, regardless of screening version, were assessed for the six SDOH domains we analyzed (financial strain, food insecurity, housing instability, transportation issues, social isolation, and health literacy).

Financial strain was measured by the frequency of positive responses to questions about challenges in paying for basic needs, such as food, housing, medical care, and heating. Food insecurity was measured using the 2-item Hunger Vital Sign (26) which focused on worrying about running out of food and running out of food over the past 12 months. Housing instability was assessed using the frequency of moves, inability to pay rent or mortgage, and homelessness during the past 12 months. Social isolation was initially measured using three questions about isolation from others, lack of companionship, and feeling left out (2021–2023); it was subsequently reduced to one question that measured feeling isolated from others. Transportation needs were measured using two questions: whether the lack of transportation as a barrier to accessing medical care, and whether it was a barrier to engaging in activities related to work and daily life. Literacy was measured as needing help with instructions and other materials from a doctor or pharmacy. Each variable was treated as a binary (yes/no) measure for the presence or absence of need in that domain. A complete list of SDOH screening questions is provided in Appendix 1, including the older and newer questions for social isolation. For all patients, we selected SDOH reported during the first OB visit as a marker of exposure around the time of conception, reflecting the embodiment theory as described by Kramer and colleagues.

### Sociodemographic characteristics

Maternal covariates included race, ethnicity, age, and type of insurance, all of which were documented in the EHR and verified by clinic staff at the time of the first prenatal intake. Rurality was defined based on patient ZIP codes and associated Rural–Urban Commuting Area (RUCA) clusters (27). RUCA codes use population density and commuting patterns to define metropolitan, micropolitan, small town, and rural areas. We created three categories:

1. Urban: Metropolitan areas with urban core populations ≥50,000.
2. Micropolitan/Small Town: Micropolitan areas with urban clusters of 10,000–49,999 population, and small towns with urban clusters of 2,500-9,999.
3. Rural: Areas with no urban cluster or with populations <2,500.

We combined micropolitan areas with small towns as both represent non-urban contexts with limited public infrastructure (e.g., public transportation, specialized services) compared to urban areas, but maintain some population density and commercial centers, including grocery stores and food pantries that distinguish them from entirely rural areas. In our service area, micropolitan centers include New Hampshire’s three largest cities (populations 44,000-115,000), which host three of our four OB-GYN practices. The separate reporting of these three categories allows examination of SDOH across the urban–rural continuum.

### Study population and eligibility criteria

We included pregnant individuals who initiated prenatal care at one of four OB-GYN clinical sites in our health system between December 2021 and December 2023; who completed SDOH screening at their first prenatal care visit; and who had documentation of their screening responses in their EHR. We excluded patients who initiated prenatal care outside of this observation period or who declined to or did not complete SDOH screening. To ensure each patient contributed data only once and to capture SDOH needs at the earliest phase of pregnancy, we analyzed only the first screening for all patients.

### Screening coverage and potential selection bias

Across all four clinical sites, 73% of patients initiating prenatal care completed SDOH screening during the study period, with site-specific rates ranging from 64–81%. Screening non-completion was primarily related to operational factors rather than systematic patient characteristics: workflows varied by site due to early implementation delays, which affected capture rates that were documented in our qualitative work (17). Patients requiring spoken language interpretation were able to complete screeners with an interpreter, facilitated by clinic staff. Nevertheless, we cannot rule out potential selection bias if patients with the most severe social needs were less likely to initiate prenatal care at all, or if certain demographic groups systematically declined screening or if screening was omitted by staff (e.g., patients with limited English proficiency, or patients with difficulty reading). This represents an important limitation of our analysis.

### Missing data

A small proportion of patients (*n* = 207; 7.1%) had some degree of missing sociodemographic information, including race, ethnicity, ZIP code, and/or insurance type. We retained these patients in our sample because our analysis focused on describing SDOH prevalence rather than conducting adjusted analyses controlling for demographic covariates. Missing data was highest for insurance type (*n* = 87; 3.9%), which may represent uninsured patients who had not yet established coverage at the time of screening. The missing data may lead to a slight underestimation of the true distribution of need by insurance status in the SDOH groups. However, because we did not conduct multivariable analyses adjusting for these covariates, the impact of missing data on our findings is limited.

### Analysis

We report descriptive statistics with standardized differences to assess balance across these SDOH comparison groups, with differences of an absolute value of <0.1 indicating balance across groups (28). We used standardized differences to assess balance across SDOH groups rather than statistical significance tests, as our goal was to describe meaningful differences across different scales rather than test hypotheses. Standardized differences are particularly useful in descriptive studies because they quantify the magnitude of differences independent of sample size. Following Cohen’s conventions, we interpreted absolute standardized differences of <0.1 as indicating balance between groups, 0.2 as small effects, 0.5 as medium effects, and 0.8 as large effects (29). We bolded values ≥0.2 in Table 1 to highlight clinically meaningful differences in patient characteristics across SDOH groups.

**Table 1:** Patient characteristics by SDOH count (*N* = 2,222 patients).

Patient characteristic	No SDOH *n* = 1851 (83.3%)	1 SDOH *n* = 198 (8.9%)	Standardized difference (None vs. 1 SDOH)	2 + SDOH *n* = 173 (7.8%)	Standardized difference (None vs. 2 + SDOH)
Mean age, years (SD)	30.7 (5.4)	29.2 (6.2)	**0.258**	27.8 (6.2)	**0.499**
Race, *n* (%)					
White	1,661 (89.7%)	177 (89.4%)	0.011	155 (89.6%)	0.005
Black	47 (2.5%)	5 (2.5%)	0.001	7 (4.0%)	−0.085
Asian	70 (3.8%)	9 (4.5%)	−0.038	4 (2.3%)	0.086
None of the above	61 (3.3%)	4 (2.0%)	0.079	5 (2.9%)	0.023
Missing	12 (0.6%)	3 (1.5%)	−0.084	2 (1.2%)	−0.054
Ethnicity, *n* (%)					
Non-Hispanic	1719 (92.9%)	184 (92.9%)	0.015	160 (92.5%)	0.015
Hispanic	77 (4.1%)	10 (5.1%)	−0.075	10 (5.8%)	−0.075
Missing	55 (3.0%)	4 (2.0)	0.082	3 (1.7%)	0.082
Rurality *n* (%)					
Urban	417 (22.5%)	8 (4.0%)	**0.566**	12 (6.9%)	**0.451**
Micropolitan/ Small Town	946 (51.1%)	142 (71.7%)	**−0.433**	121 (69.9%)	**−0.393**
Rural	440 (23.8%)	47 (23.7%)	0.001	37 (21.4%)	0.057
Missing	48 (2.6%)	1 (0.5%)	0.170	3 (1.7%)	0.059
Payer (*n*%)					
Commercial	1,377 (74.4%)	122 (61.6%)	**0.276**	48 (27.8%)	**1.055**
Public	413 (22.3%)	63 (31.8%)	**−0.215**	112 (64.7%)	**−0.947**
Missing	61 (3.3%)	13 (6.6%)	−0.151	13 (7.5%)	−0.187
Site of care *n* (%)					
Academic center	1,215 (65.6%)	170 (85.9%)	**−0.485**	147 (85.0%)	**−0.460**
Ambulatory Practice	636 (34.4%)	28 (14.1%)	0.485	26 (15.0%)	0.460

Absolute values >0.2 indicate clinically meaningful differences in groups.

To examine the co-occurrence of SDOH in the study population, we generated an UpSet plot using the UpSetR statistical software package in R to visualize set intersections and their frequencies (30, 31). Input data consisted of binary indicators for each SDOH type, with intersections calculated based on individuals experiencing multiple SDOH risk factors. The plot displays the frequency of each unique combination of factors, with bar charts summarizing the overall prevalence of each risk factor type. This visualization facilitates the identification of common co-occurrence patterns within the study population. Further analysis of outcomes was not undertaken as we have incomplete birth outcomes for our prenatal care patients who deliver at hospitals outside our system. This analysis will be forthcoming once we have complete data on perinatal outcomes and is noted in the limi.

### Ethical considerations

This analysis received IRB approval as non-human subjects research, as SDOH screening was implemented as standard clinical care rather than a research study, i.e., it was designated as quality improvement. However, informed participation principles were maintained through several mechanisms. First, screening was part of the routine prenatal intake and patients could choose to answer any or all questions without consequence to their care. The clinic response to identified needs includes an on-site food pantry services and referrals to embedded community health workers. Second, patients could choose whether to accept referrals or resource information based on their screening responses without impacting their clinical care. Third, the brief nature of the screening (14 questions) and integration into existing intake workflows minimized burden on patients and staff.

## Findings

Of 3,044 pregnant individuals initiating prenatal care during the study period, 2,222 (73%) completed SDOH screening at their initial prenatal care visit (Table 1). Aggregating across sites, the prevalence of having only one social need at the start of prenatal care was 8.9%, while the prevalence of having two or more needs was 7.8%, resulting in 16.7% of patients reporting at least one social need. Patients with any SDOH needs were younger, on average, than those without identified needs, with the lowest mean age observed among those with two or more needs. Most patients identified as White (>89%) and non-Hispanic (>92%). Compared to individuals with no identified social needs, a higher proportion of those with at least one unmet social need lived in micropolitan or small towns than urban or rural areas. No differences in SDOH groups were observed for rurality. Compared to individuals with no identified social needs, those with two or more social needs also had much higher rates of public health insurance coverage (22.3% vs. 64.7%). However, nearly 11% of commercially insured patients also reported having one or more social needs, demonstrating that insurance status incompletely captures patient needs and underscores the American reality that even commercially insured patients with enough income to exclude them from Medicaid coverage may experience resource gaps. Notably, patients with SDOH were more likely to enroll in prenatal care at the rural academic medical center than at the other ambulatory more urban practices. A larger proportion of patients with unmet needs were missing data on insurance type, likely an indicator of being uninsured.

The most common SDOH experienced by prenatal patients was financial stress, followed by social isolation, food insecurity, and housing instability (Table 2). For those experiencing only one SDOH, social isolation was the most prevalent (38.9%), however, a comparable proportion of patients with two or more needs also reported feeling isolated. Three of four people with multiple needs worried about finances, and nine of 10 who experienced food insecurity experienced at least one other concurrent need.

**Table 2:** SDOH prevalence among pregnant patients.

SDOH domain	1 SDOH (*n* = 198)	2 + SDOH (*n* = 173)	Any SDOH (*n* = 371)
Financial stress	55 (27.8%)	130 (75.1%)	185 (49.9%)
Isolation	77 (38.9%)	74 (42.8%)	151 (40.7%)
Food insecurity	11 (5.6%)	117 (67.6%)	128 (34.5%)
Housing	30 (15.2%)	97 (56.1%)	127 (34.2%)
Literacy	20 (10.1%)	24 (13.9%)	44 (11.9%)
Transport	5 (2.5%)	38 (22.0%)	43 (11.6%)

**Figure 2:**
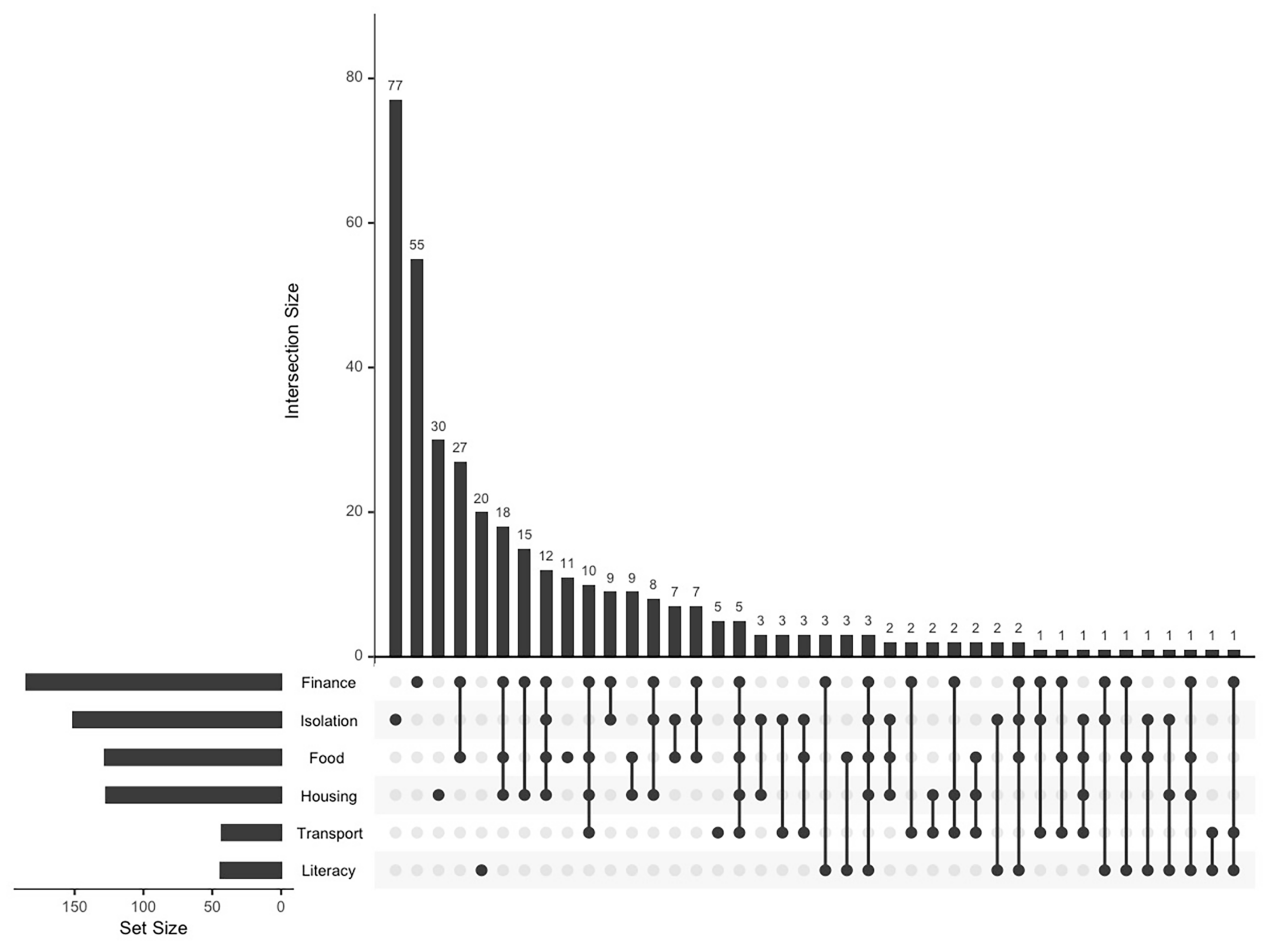
Upset plot of the intersection of social determinants of health among pregnant women.

Figure 2 displays an UpSet plot illustrating the intersections among the six SDOH domains of the final screening instrument. The horizontal bars in the lower panel represent the total number of individuals experiencing each SDOH, while the vertical bars quantify the number of individuals affected by each unique combination of SDOH. The matrix in the center highlights specific intersections between SDOH domains. Filled dots indicate which domains are included in each intersection. There were no SDOH items that were unique to just one set, even if they occurred solely for some patients. For example, social isolation and financial stress were exclusively experienced by 77 and 55 patients, respectively, but both needs were also experienced in combination with several other domains (represented by single dots connected to other determinants with a line). Notably, while social isolation appeared as a stand-alone need for 77 patients (20.8% of those with any SDOH), it also appeared in combination with nearly every other SDOH domain (e.g., 12 patients experienced isolation in combination with financial stress, food insecurity, and housing instability). The clustering of food insecurity, housing instability, and financial stress (*n* = 18 with all three) does not simply reflect three separate independent problems but signals communities characterized by multiple simultaneously-operating constraints: limited affordable housing stock, food deserts, inadequate public transportation to reach resources, and constrained economic opportunity (6th vertical bar from the left). Nearly half of patients with any social need (*n* = 179) struggled with some combination of food, housing, financial stress, or social isolation, a pattern consistent with residing in communities where systematic resource scarcity creates constraints across multiple domains. The relatively high number of patients experiencing four or more concurrent needs (*n* = 33, 8.9% of those with any SDOH) highlights a small but extremely high-risk subgroup. Transportation issues, while less prevalent overall (11.6%), appeared almost exclusively among patients with other needs (87% had 2 or more needs). This pattern exemplifies how community-level infrastructure constraints compound material hardship.

## Discussion

We found that nearly as many patients experienced co-occurring SDOH needs as reported any single social determinant. These findings provide empirical evidence of how community-level constraints manifest as co-occurring unmet social needs among pregnant individuals. The successful implementation of universal SDOH risk screening at designated prenatal care visits across our health system provided an opportunity to describe the prevalence and intersection of SDOH needs during early pregnancy. Unsurprisingly, financial stress was the most frequently reported SDOH, followed by social isolation, food insecurity, and housing instability. Among those with only one expressed need, social isolation was most prevalent, demonstrating potential vulnerability even among prenatal patients with financial stability. Dominant intersections were observed between food insecurity and financial stress, housing instability and financial stress, and food insecurity and housing instability. The overall picture for this subset of patients is sobering, indicating multiple needs, each associated with implications for perinatal health. Notably, our findings demonstrate co-occurrence among social needs, with no single domain occurring in isolation, and nearly half of affected patients experiencing multiple concurrent challenges related to basic subsistence and social support. The most frequent intersection of food insecurity with financial stress suggests that food access problems often stem from broader economic constraints rather than food system factors alone. This pattern reflects how the lack of economic opportunities such as limited living-wage employment and inadequate social safety nets may contribute to constraints in food access. Interventions addressing only food provision (e.g., food pantry referrals) may provide temporary relief but do not address the underlying economic gaps that create food insecurity (32, 33).

Considerable variation exists in the published literature regarding which SDOH are measured, how they are measured, and for which populations. The screening tool utilized by this health system did not include race, ethnicity, payor, preferred language, educational attainment, nativity, or immigration status as SDOH (although the first four characteristics are documented elsewhere in the EHR), focusing instead on proximal determinants that could be directly mitigated through interventions by doulas and/or community health workers rather than the upstream structural injustice leading to them. Our analysis focused specifically on SDOH needs in the prenatal care population rather than postpartum. Thus, there were parallels and differences between our findings and prior research.

For example, a 2015 study of pregnant Medicaid-insured women in North Carolina, screening for housing instability revealed a prevalence of 6.2% (34), while we found a prevalence of 14.0% among Medicaid beneficiaries, potentially reflecting geographic as well as temporal variation. Investigators in California using relevant International Classification of Disease codes rather than direct screening for SDOH reported a much lower prevalence of unmet social needs for publicly (0.11%) and privately (0.014%) insured mothers and did not report overlapping needs (35). In contrast, in an antenatal care population in Georgia, investigators found that a little over a third of women screened positive for at least one SDOH during the first trimester, greater than our finding of one in five women (36). Notably, the Georgia cohort was predominantly non-Hispanic Black and publicly insured, in contrast to our predominantly White sample with mixed insurance types; however, in parallel to our study, women experiencing any SDOH need were younger than those without identified needs. Finally, a recent study documenting birth outcomes across Chicago reported a prevalence of SDOH needs of 4.2% among all pregnant patients, which is lower than our overall estimates (37). However, the authors reported comparable estimates to ours for those insured through Medicaid. We did not identify other studies reporting multiple individual needs during pregnancy, although others have explored the overlay of individual and neighborhood or area level SDOH characteristics (38, 39). Our study, therefore, uniquely captures an intersectional dimension of social risk during pregnancy that has been explored by few investigators to date.

The higher prevalence of social isolation compared to other needs was unexpected. This had not been shared explicitly by patients in our qualitative work, although they did report intermittent support from family, friends, and neighbors as being helpful, even if unpredictable. Social isolation is infrequently measured in the published literature as a social determinant, despite evidence linking limited social support during pregnancy with increased risk of smoking, poor mental health, low birthweight, and preterm birth (40). Among our patients, the prevalence of social isolation for those with two or more needs also raises the concern that those who lack resources such as food and transportation or who are younger are also isolated from family and community, who might otherwise help to mitigate the impact of unmet needs. This pattern suggests two distinct populations: those who were socially isolated but otherwise resourced, and those with multiple needs who also lacked social support networks. The latter group may reside in communities characterized by concentrated poverty, where informal social networks might exist but are themselves resource-constrained, limiting capacity for mutual aid. These groups may require different intervention approaches: the first group benefits from social connection programs, while the second requires both resource navigation and social support, along with efforts to address the economic constraints affecting their entire community.

### Clinical and research implications

Universal and comprehensive SDOH screening can play a pivotal role in identifying underlying factors that contribute to disparities in maternal health outcomes potentially missed by single-item and/or targeted screening (41). By systematically screening our perinatal population, we were able to determine the extent of social risk faced by individual patients during a critical, early point in their prenatal care and to intervene when supportive services could have a greater impact on pregnancy outcomes. Medicaid insurance alone should not be considered a surrogate measure of need, as commercially insured individuals also reported having multiple social needs during pregnancy (42). Universal screening ensures that privately insured patients who might not be chosen for targeted screening are not missed in social risk assessment. As one author put it, “if you do not ask, you will not know” (43).

Accrediting bodies and payors are increasingly mandating SDOH screening based on the established evidence that screening has significant clinical implications when patients are effectively linked to community resources. Identifying SDOH needs early in pregnancy not only ensures timely support but also helps establish trust that is essential in meeting patient needs well into the postpartum period, especially for those struggling with behavioral health comorbidities such as substance use or mental health challenges (44, 45). Moreover, we found that intersecting and overlapping needs were prevalent and therefore, must be acknowledged and addressed. Prior research has demonstrated that food insecurity during pregnancy is associated with gestational diabetes, hypertensive disorders, preterm delivery, and low birth weight, while housing instability correlates with preterm labor and severe maternal morbidity (6–10). When these needs co-occur with financial stress, as we observed in the majority of patients with multiple SDOH, the cumulative burden may amplify adverse outcomes through multiple pathways: chronic stress activation, inadequate nutrition, delayed prenatal care due to transportation barriers, and reduced capacity to manage pregnancy-related complications. However, these individual-level mechanisms are also likely to occur within constrained community environments that systematically limit access to resources necessary for healthy pregnancy.

As with screening for any high risk condition, positive screening results must be connected to follow-up care. Our community case study suggests that nearly half of our patients with a social need have at least one additional need, likely requiring complex or multiple interventions across contexts. Research is necessary to study the effectiveness of connecting people to community resources in addressing one or more unmet social needs, including how patients navigate multiple overlapping needs and are able to access supportive services while also participating in maternal care.

Notably, our findings demonstrate that unmet social needs rarely occur in isolation, and nearly half of affected patients experienced multiple concurrent challenges related to basic subsistence and social support, patterns consistent with residing in communities shaped by spatial and social stratification. For patients with multiple needs, a single intervention may be insufficient; for example, if they continue to face food and financial insecurity generated by their community environment. Our work supports the need for comprehensive case management that addresses multiple domains simultaneously, although long term solutions will require policy interventions that modify the economic context. As noted, rural areas are characterized by systematic healthcare infrastructure constraints, i.e., fewer providers, greater distances to specialty care, limited public transportation, that create barriers to prenatal care access independent of individual characteristics. The combination of economic and geographic risk produces particularly constrained environments: rural, low-income communities where employment opportunities are limited, wages are low, distances to services are great, and transportation infrastructure is minimal (46, 47).

### Limitations

A significant limitation of this analysis is the absence of inferential statistics exploring associations between constrained community environments (as indicated by SDOH) and adverse clinical outcomes such as obstetric complications or birth outcomes. Nearly half of patients receiving prenatal care at our sites delivered at hospitals outside our health system, limiting our capacity to incorporate birth outcomes in this retrospective analysis. As stated above, our team continues to work toward a more complete dataset enabling an evaluation that will include outcomes in the context of social needs, clinical risk factors, and structural determinants of health. The current study should therefore be understood as a descriptive analysis of SDOH prevalence and co-occurrence patterns as indicators of constrained environments, rather than an examination of associations with health outcomes. We plan to rigorously study the association between unmet social needs and birth outcomes, and longitudinal changes in social needs over the course of pregnancy as a result of interventions during pregnancy into the postpartum period.

While some components of our screening tool have strong psychometric properties (e.g., the 2-item Hunger Vital Sign), other domains were assessed using single items that may not capture the full construct of complex social phenomena and may be more susceptible to measurement error or varying interpretation across respondents. Most notably, social isolation was measured with a single question (‘Do you feel isolated from others?’) after the tool was shortened, rather than the original three-item scale. However, our findings in this domain were consistent with qualitative findings regarding limited social support in our prior research. Additionally, our screening tool captures SDOH but does not systematically measure access to community-level resources. This creates an incomplete picture of community environments, emphasizing deficits while potentially missing strengths which might mitigate harm. Linking individual screening data to community-level indicators (as proposed in the Kramer framework) would provide more complete understanding of how community constraints generate individual unmet needs.

With regard to generalizability, our findings may not apply to populations with different racial and ethnic composition, immigration status, or large urban settings with different constrained environments and social service opportunities. Our sample’s racial homogeneity limits our ability to examine how spatial and social stratification along racial lines produces differential exposure to constrained environments. The Kramer framework emphasizes that residential segregation by race is a fundamental social determinant systematically producing constrained environments for Black and Hispanic populations through historical processes like redlining and ongoing housing market discrimination (21). Our predominantly White sample cannot illuminate these racial stratification processes, representing an important limitation for understanding health equity.

We were unable to compare demographic characteristics between groups who completed and did not complete the screening, potentially introducing bias into our sample as patients experiencing the most severely constrained environments may have been least likely to access prenatal care and/or complete screening. Finally, the current study does not explore referral acceptance, connection rates, service utilization, or need resolution following screening. This represents a critical gap to be addressed in future research in understanding the effectiveness of screening-to-intervention pathways and whether individual-level interventions can meaningfully address resource needs generated by constrained community environments.

## Conclusion

This case study supports the importance of embedding standardized SDOH screening in prenatal care to establish the prevalence and intersection of social risk in the perinatal population. Our descriptive findings, interpreted through a maternal health equity framework, focused on community-level constraints and provide foundational evidence of how unmet social needs cluster in patterns reflecting spatial and social population stratification. While responding to individual needs is always necessary, patients with overlapping needs are especially vulnerable to adverse birth outcomes. Clinical practice should consider a patient-centered approach capable of universal identification of SDOH needs, understanding how needs intersect, and delivery of multi-levels responses to meeting intersecting social needs. Clinical interventions are needed at all levels, from individual resource provision to advocacy for policies that address residential segregation, economic inequality, and rural disinvestment and their impact on pregnant and birthing people in the United States.

## Data Availability

The data analyzed in this study is subject to the following licenses/restrictions: the data are not included as they are protected patient information. Requests to access these datasets should be directed to AI_ResearchData_Information@hitchcock.org.
