# Supplementary material for: Characterizing intersecting social determinants of health during pregnancy: a descriptive cross-sectional analysis from a northern New England health system
Source: Front Med (Lausanne). 2025 Dec 17;12:1658735. doi: 10.3389/fmed.2025.1658735 (PMC12753359; doi:10.3389/fmed.2025.1658735)
Supplement: Supplementary file 2 [file Table_2.doc]

Appendix 2. The flow of participants through screening

Initiated prenatal care

(n = 3,044)

Excluded (n 822)

- Refused screening
- Not offered screening
- Did not complete screening

2,222 completed screening at first prenatal care visit

# 
